# IMproving Preclinical Assessment of Cardioprotective Therapies (IMPACT) criteria: guidelines of the EU-CARDIOPROTECTION COST Action

**DOI:** 10.1007/s00395-021-00893-5

**Published:** 2021-09-13

**Authors:** Sandrine Lecour, Ioanna Andreadou, Hans Erik Bøtker, Sean M. Davidson, Gerd Heusch, Marisol Ruiz-Meana, Rainer Schulz, Coert J. Zuurbier, Péter Ferdinandy, Derek J. Hausenloy, on behalf of the European Union-CARDIOPROTECTION COST ACTION CA16225, Pavle Adamovski, Ioanna Andreadou, Saime Batirel, Monika Barteková, Luc Bertrand, Christophe Beauloye, David Biedermann, Vilmante Borutaite, Hans Erik Bøtker, Stefan Chlopicki, Maija Dambrova, Sean Davidson, Yvan Devaux, Fabio Di Lisa, Dragan Djuric, David Erlinge, Ines Falcao-Pires, Péter Ferdinandy, Eleftheria Galatou, Alfonso Garcia-Sosa, Henrique Girao, Zoltan Giricz, Mariann Gyongyosi, Derek J Hausenloy, Donagh Healy, Gerd Heusch, Vladimir Jakovljevic, Jelena Jovanic, George Kararigas, Risto Kerkal, Frantisek Kolar, Brenda Kwak, Przemysław Leszek, Edgars Liepinsh, Jacob Lonborg, Sarah Longnus, Jasna Marinovic, Danina Mirela Muntean, Lana Nezic, Michel Ovize, Pasquale Pagliaro, Clarissa Pedrosa Da Costa Gomes, John Pernow, Andreas Persidis, Søren Erik Pischke, Bruno Podesser, Ines Potočnjak, Fabrice Prunier, Tanya Ravingerova, Marisol Ruiz-Meana, Alina Serban, Katrine Slagsvold, Rainer Schulz, Niels van Royen, Belma Turan, Marko Vendelin, Stewart Walsh, Nace Zidar, Coert Zuurbier, Derek Yellon

**Affiliations:** 1 Department of Medicine, Hatter Institute for Cardiovascular Research in Africa, University of Cape Town, Cape Town, South Africa; 2 Laboratory of Pharmacology, Faculty of Pharmacy, National and Kapodistrian University of Athens, Athens, Greece; 3 Department of Cardiology, Aarhus University Hospital, Aarhus N, Denmark; 4 The Hatter Cardiovascular Institute, University College London, London, UK; 5 Institute for Pathophysiology, West German Heart and Vascular Center, University of Duisburg-Essen, Essen, Germany; 6 Cardiovascular Diseases Research Group, Vall d’Hebron Institut de Recerca (VHIR), Vall d’Hebron Hospital Universitari, Vall d’Hebron Barcelona Hospital Campus, Barcelona, Spain; 7 Institute for Physiology, Justus-Liebig University Giessen, Giessen, Germany; 8 Laboratory of Experimental Intensive Care Anesthesiology, Department Anesthesiology, Amsterdam Cardiovascular Sciences, Amsterdam UMC, University of Amsterdam, Amsterdam, The Netherlands; 9 Department of Pharmacology and Pharmacotherapy, Semmelweis University, Budapest, Hungary; 10 Pharmahungary Group, Szeged, Hungary; 11 Cardiovascular & Metabolic Disorders Program, Duke-National University of Singapore Medical School, 8 College Road, Singapore, 169857 Singapore; 12 National Heart Research Institute Singapore, National Heart Centre, Singapore, Singapore; 13 Yong Loo Lin School of Medicine, National University Singapore, Singapore, Singapore; 14 Cardiovascular Research Center, College of Medical and Health Sciences, Asia University, Taichung, Taiwan

**Keywords:** Cardioprotection, Drug development, Ischemia, Reperfusion, Infarction

## Abstract

Acute myocardial infarction (AMI) and the heart failure (HF) which may follow are among the leading causes of death and disability worldwide. As such, new therapeutic interventions are still needed to protect the heart against acute ischemia/reperfusion injury to reduce myocardial infarct size and prevent the onset of HF in patients presenting with AMI. However, the clinical translation of cardioprotective interventions that have proven to be beneficial in preclinical animal studies, has been challenging. One likely major reason for this failure to translate cardioprotection into patient benefit is the lack of rigorous and systematic in vivo preclinical assessment of the efficacy of promising cardioprotective interventions prior to their clinical evaluation. To address this, we propose an in vivo set of step-by-step criteria for **IM**proving **P**reclinical **A**ssessment of **C**ardioprotective **T**herapies (‘IMPACT’), for investigators to consider adopting before embarking on clinical studies, the aim of which is to improve the likelihood of translating novel cardioprotective interventions into the clinical setting for patient benefit.

## Introduction

Acute myocardial infarction (AMI) and the heart failure (HF) that may follow are among the leading causes of death and disability worldwide. For patients presenting with an acute ST-segment elevation myocardial infarction (STEMI), the treatment priority for limiting myocardial infarct (MI) size and preventing the onset of HF, is timely myocardial reperfusion by primary percutaneous coronary intervention (PPCI). Despite a decline in early mortality, the number of STEMI patients going on to develop post-infarct HF remains high [34, 36]. As such, there is an urgent need to discover novel therapeutic interventions that can be applied as an adjunct to PPCI to reduce MI size and prevent post-infarct adverse left ventricular (LV) remodelling [19, 20]. However, the translation into the clinical setting of novel cardioprotective interventions that have been claimed to be effective in experimental animal studies of MI has been extremely challenging and largely disappointing, leading to much discussion in recent literature [5, 10, 17, 18, 21, 23].

Importantly, the endpoints in experimental and clinical studies differ. The most robust primary endpoint in experimental studies on cardioprotection is infarct size [3], although coronary microvascular injury is also increasingly recognized as a manifestation of acute myocardial ischemia/reperfusion injury (IRI) and thus a target of cardioprotection [11, 15, 16]. Although infarct size and coronary microvascular obstruction are major determinants of patients´ prognosis [7, 35], they are only surrogate endpoints when compared to the primary clinical endpoints of mortality and/ or hospitalization for HF. Thus, not only the endpoints per se but also the time frame over which these endpoints are assessed differ between experimental and clinical studies.

One key reason for the failure to realize cardioprotection beyond timely coronary revascularization in the clinical arena may be the lack of rigorous and systematic pre-clinical in vivo efficacy testing of novel cardioprotective interventions, the consequence of which has been the premature clinical evaluation of treatments with inconsistent and less-than-robust cardioprotective effects. To address this, experts in the field of cardioprotection and the European Union-CARDIOPROTECTION COST Action CA16225 [12] have joined forces to establish step-by-step criteria for **IM**proving **P**reclinical **A**ssessment of **C**ardioprotective **T**herapies (IMPACT). We anticipate that adoption of these criteria will increase the likelihood of successful clinical translation of cardioprotective interventions showing promise in preclinical animal studies.

The focus of the IMPACT criteria will be on the in vivo preclinical assessment of efficacy of cardioprotective drugs and performance of cardioprotective devices. Safety and regulatory issues pertaining to cardioprotective drug and device development are beyond the scope of the IMPACT criteria and will not be dealt with in this document.

## IMPACT criteria for in vivo preclinical assessment of cardioprotective interventions

**Fig. 1:**
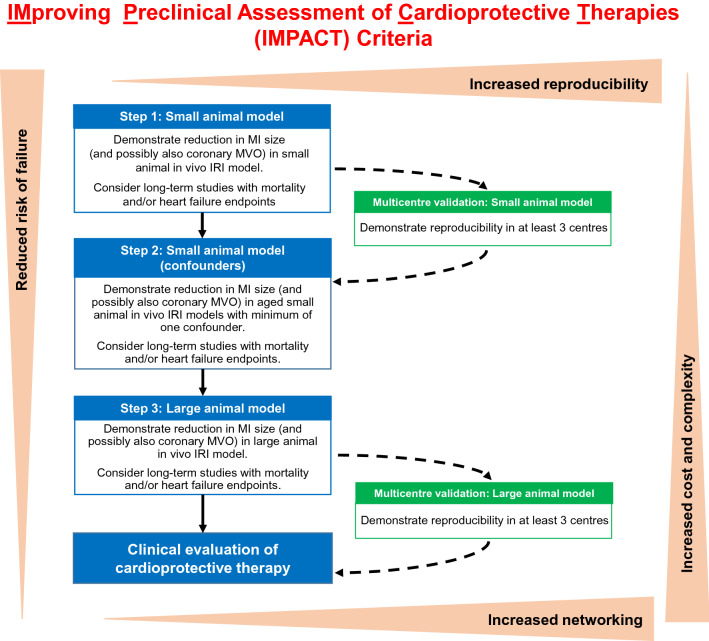
Overview of IMPACT criteria for improving the preclinical evaluation of novel cardioprotective interventions. *IRI* ischaemia/reperfusion injury, *MI* myocardial infarct, *MVO* microvascular obstruction

To provide a rigorous and systematic approach to the in vivo preclinical evaluation of efficacy and performance of novel cardioprotective interventions, we propose a step-by-step approach for in vivo validation (the IMPACT criteria) in both small and large animal models prior to clinical evaluation (Fig. 1). Although we appreciate that not all steps are relevant for the assessment of both drugs and medical devices (given the impact of study costs, complexity, and various regulatory requirements), completing more steps is likely to correlate with a reduced risk of failure in the clinical translation of novel cardioprotective interventions. For each step, we propose the *minimum IMPACT criteria* that need to be met to validate a particular step and the *desirable IMPACT criteria* which, when adhered to, may further reduce the risk of translation failure. Finally, in our IMPACT criteria, collaborations and the formation of networks between research groups are encouraged to improve the rigor and reproducibility of in vivo pre-clinical efficacy and performance studies evaluating novel cardioprotective interventions. Published practical general [6, 24, 32] and cardioprotection-specific [3, 28] guidelines or recommendations for rigor and reproducibility in preclinical studies should be followed together with the current proposed IMPACT criteria in the preclinical evaluation of novel cardioprotective interventions. A summary of the key recommendations is shown in Table 1.

**Table 1:** Summary of key recommendations for cardioprotection study design

Study design variable	General recommendations for cardioprotection study design
Inclusion and exclusion criteria	These must be specified in advance and reported as transparently and as detailed as possible
Sample size	This should be determined in advance to required effect size and local data on variability of infarct size/coronary microvascular obstruction measurements
Randomization	Animals should be randomly allocated to the treatment groups to avoid bias
Blinded treatment allocation and analysis	Where possible treatment allocation should be blinded
Study endpoints	Infarct size is the gold standard primary endpoint (coronary microvascular obstruction should also be considered)
Blinded analysis	Infarct size and coronary microvascular obstruction should be assessed in a blinded fashion

### Step 1: IMPACT criteria for in vivo validation in healthy small animal models

Once a drug or treatment strategy with a medical device has been identified to be a potential cardioprotective therapy, the first step in the translation pathway is to validate its cardioprotective efficacy or performance in an in vivo small animal model of acute myocardial IRI (Table 2). This validation step can be performed using either mouse, rat or rabbit models, although the use of two of these species or alternatively two different strains is desirable to ensure its consistent effect across species/strains. At this stage, validation in one centre may be sufficient considering that subsequent steps will involve multicentre validation. We recommend that the MI model should comprise both acute myocardial ischemia and reperfusion (rather than permanent occlusion, to better represent the clinical scenario) [1], and the endpoint for cardioprotection should be infarct size relative to the area-at-risk (also, consider coronary microvascular obstruction as another important endpoint) with a minimum of 2 h of reperfusion and preferably 24 h of reperfusion [3]. However, measurement of infarct size at 2 h by tetrazolium staining may not be as robust as measuring it at 24 h of reperfusion. It is desirable to demonstrate benefit of the intervention after at least 28 days (by assessing scar size relative to LV mass and LV remodelling). We acknowledge that scar size at this time-point, LV function and remodelling are confounded by other variables beyond infarct size and coronary microvascular obstruction with a longer reperfusion time but this is also true in the clinical setting which we wish to predict with preclinical data.

**Table 2:** IMPACT criteria for the in vivo preclinical evaluation of efficacy and performance of novel cardioprotective interventions

**Step 1: IMPACT criteria for validation in healthy small animal models**
*Minimum criteria:*
Validation in one species (e.g.: mouse, rat or rabbit)
Validation in a single centre
Acute IRI model (minimum of 2 h and preferably 24 h of reperfusion)
Infarct size relative to area-at-risk and possibly also coronary microvascular obstruction
*Desirable criteria:*
Validation in 2 different species/strains
Chronic IRI model (at least 28 days of reperfusion)
Infarct size and LV remodelling (at least 28 days of reperfusion)
**Step 1a**: **IMPACT criteria for multicentre validation in healthy small animal models**
*Minimum criteria:*
Validation in at least one species
Validation in at least 3 centres
Acute IRI model (minimum of 2 h and preferably 24 h of reperfusion)
Infarct size and possibly also coronary microvascular obstruction
*Desirable criteria:*
Validation in 2 different species/strains
Chronic IRI model (at least 28 days of reperfusion)
Infarct size and LV remodelling (at least 28 days post-infarction)
**Step 2: IMPACT criteria for validation in small animal models with confounders**
*Minimum criteria:*
Validation in the presence of at least one confounder (e.g. age, diabetes mellitus, P2Y_12_ inhibitor)
Acute IRI model (minimum of 2 h and preferably 24 h of reperfusion)
Infarct size and possibly also coronary microvascular obstruction
*Desirable criteria:*
Validation in both male and female animals
Validation in the presence of two or more confounders
Chronic IRI model (at least 28 days of post-infarction)
Infarct size and LV remodelling (at least 28 days post-infarction)
**Step 3: IMPACT criteria for validation in large animal models**
*Minimum criteria:*
Validation in one species (e.g.: pig)
Validation in a single centre
Acute IRI model (minimum of 2 h and preferably 72 h of reperfusion)
Infarct size and possibly also coronary microvascular obstruction
*Desirable criteria:*
Validation in both male and female animals
Chronic IRI model (at least 3 months post-infarction)
Infarct size and LV remodelling (at least 3 months post-infarction)
Assessment in animals with a co-morbidity
**Step 3a: IMPACT criteria for multicentre validation in large animal models**
*Minimum criteria:*
Validation in at least 3 centres
Acute IRI model (minimum of 2 h and preferably 72 h of reperfusion)
Infarct size and possibly also coronary microvascular obstruction
*Desirable criteria:* Validation in 2 different species/strains Chronic IRI model (at least 3 months post-infarction) Infarct size and LV remodelling (at least 3 months post-infarction)

### Step 1a (desirable): IMPACT criteria for multicentre in vivo validation in healthy small animal models

Once the cardioprotective efficacy or performance of the cardioprotective intervention has been optimized in a single-centre, small animal IRI model, a multicentre validation study in a minimum of 3 centres in at least one species (typically rat or mouse) should strongly be considered to validate study reproducibility (Table 2). This can be achieved by independent research centres or by the formation of a network of research centres working in partnership to undertake small animal IRI studies to evaluate the cardioprotective intervention in a blinded fashion using standardized protocols and centralized core lab analysis of infarct size and/or coronary microvascular obstruction.

The concept of a multicentre in vivo preclinical evaluation of cardioprotective interventions was first tested in 2010, with the National Heart, Lung, and Blood Institute (NHLBI)-funded, Consortium for preclinicAl assESsment of cARdioprotective interventions (CAESAR) research network of 3 sites with capabilities for performing acute myocardial IRI studies in mice, rabbits and pigs [2, 25, 30, 34]. The network encompassed the principles of randomization, investigator blinding, a priori sample size determination and exclusion criteria, appropriate statistical analyses, assessment of reproducibility, and core lab analysis of histology and biomarkers. Although the CAESAR consortium demonstrated cardioprotection with ischemic preconditioning [25], it failed to reproduce cardioprotection with pharmacological agents which had been previously shown to be cardioprotective in single-site studies such as nitrite [29] and sildenafil [27]. Although the consortium is no longer functioning, it illustrates the utility of a multicentre network for preclinical evaluation of novel cardioprotective interventions. The EU-CARDIOPROTECTION COST Action [12] is currently establishing a small animal research network to undertake multicentre, pre-clinical evaluation of novel cardioprotective interventions in mice and rat models of acute myocardial IRI. Initial validation of the effectiveness of the research network will be undertaken using ischemic preconditioning. We propose to utilize a rigorous, standardized, multicentre study protocol with a centralized assessment of infarct size as described in the NHLBI-CAESAR.

### Step 2: IMPACT criteria for in vivo validation in small animal model with confounders

A variety of factors have been shown to attenuate the efficacy of cardioprotective interventions in pre-clinical models, although the evidence for this occurring in clinical cardioprotection studies is limited [26]. These confounding factors include age, sex, and certain co-morbidities (such as diabetes, hypertension or dyslipidaemia) and co-medications often used during clinical procedures (platelet P2Y_12_ inhibitors, heparin, morphine, sedatives, anaesthetics) and more chronic care (such as anti-diabetic medications, statins and nitrates) are likely to reduce cardioprotective efficacy by impacting on intrinsic cardioprotective signalling pathways [9]. Therefore, following validation in healthy animals, step 2 of the IMPACT criteria requires validation in a small animal IRI model in the presence of at least one of these confounding factors using either a mouse, rat or rabbit model with infarct size (and consider also coronary microvascular obstruction) at 2–24 h of reperfusion measured as an endpoint. The choice of confounding factor(s) will depend mainly on the mechanism of the intervention tested, available resources and facilities but we recommend that investigators consider including age, metabolic diseases like diabetes or hypercholesterolemia and/or administration of a platelet P2Y_12_ inhibitor. A useful approach can be to use a combination of drugs representing the clinically relevant background drugs typically administered to STEMI patients, which has been shown to affect assessment of cardioprotective interventions [13]. At this step, desirable criteria include testing sex-differences [31], multiple confounders and demonstrating cardioprotection after at least 28 days (in terms of reduced infarct size and less adverse LV remodelling).

### Step 3: IMPACT criteria for validation in large animal model

The penultimate step in the clinical translation pathway is the preclinical evaluation of the efficacy of novel cardioprotective interventions in a large animal IRI model. Therefore, step 3 of the IMPACT criteria requires validation in a large animal model, most often the pig, given its anatomic similarities and the similar temporal and spatial distribution of infarction to the human heart [22], in a single centre study with infarct size and/or coronary microvascular obstruction measured at 2–72 h reperfusion. Desirable criteria include demonstrating cardioprotection (in terms of reduced infarct size and less adverse LV remodelling) after at least 3 months of reperfusion using histology and cardiac MRI [4, 14].

### Step 3a (desirable): IMPACT criteria for multicentre validation in large animal model

The final step of validation to consider prior to clinical testing of a novel cardioprotective therapy is multicentre validation using a large animal IRI model, although costs and study logistics for such a study are challenging. This validation (ideally in pigs) should be performed with a minimum of 3 centres using a short-term (2–72 h) and/or a long-term recovery (at least 3 months) model. It should be undertaken according to pre-defined design and protocols, centralized randomization and blinded core lab analysis. Where available the use of both cardiac MRI and histology techniques to assess the infarct size and coronary microvascular obstruction is recommended. In this regard, the CIBERCV (acronym for Spanish network-center for cardiovascular biomedical research) has set up the "Cardioprotection Large Animal Platform" (CIBER-CLAP), a Spanish multicentre network of 5 research centres performing acute myocardial IRI in pigs for testing the efficacy and reproducibility of novel cardioprotective interventions. This network is currently being validated using ischemic preconditioning with mechanical coronary occlusion/reperfusion as a cardioprotective strategy [33]. Although it is challenging, testing of cardioprotective drugs in a pig model with co-morbidities would be ideal.

## Future perspectives

The IMPACT criteria have been drawn up to address the challenge of translating cardioprotective interventions into the clinical setting for patient benefit with a large number of neutral clinical cardioprotection studies. These criteria aim to improve the rigor and reproducibility of in vivo preclinical efficacy and/or performance studies for cardioprotection, by setting out step-by-step criteria required for preclinical evaluation of novel cardioprotective interventions in small and large animals. The aim of the IMPACT criteria is to increase the likelihood of translating cardioprotective interventions into the clinical setting. Adhesion to these criteria and published guidelines for rigor and reproducibility in preclinical studies on cardioprotection [3] will require a paradigm shift in the way investigators undertake cardioprotective research. Moreover, in addition to cardioprotective efficacy and performance, the evaluation of safety of novel cardioprotective drugs and medical devices (not covered in this document) will also need to be addressed [8]. Working together towards the same hypothesis by sharing expertise, knowledge and experimental models is likely to improve rigor, reproducibility and increase the chances of translating cardioprotection for the benefit of patients, although this remains to be proven.

## References

[1] Basalay MV, Yellon DM, Davidson SM (2020). Targeting myocardial ischaemic injury in the absence of reperfusion. Basic Res Cardiol.

[2] Bolli R (2021). CAESAR's legacy: a new era of rigor in preclinical studies of cardioprotection. Basic Res Cardiol.

[3] Botker HE, Hausenloy D, Andreadou I, Antonucci S, Boengler K, Davidson SM, Deshwal S, Devaux Y, Di Lisa F, Di Sante M, Efentakis P, Femmino S, Garcia-Dorado D, Giricz Z, Ibanez B, Iliodromitis E, Kaludercic N, Kleinbongard P, Neuhauser M, Ovize M, Pagliaro P, Rahbek-Schmidt M, Ruiz-Meana M, Schluter KD, Schulz R, Skyschally A, Wilder C, Yellon DM, Ferdinandy P, Heusch G (2018). Practical guidelines for rigor and reproducibility in preclinical and clinical studies on cardioprotection. Basic Res Cardiol.

[4] Brenner GB, Giricz Z, Garamvolgyi R, Makkos A, Onodi Z, Sayour NV, Gergely TG, Baranyai T, Petnehazy O, Korosi D, Szabo GP, Vago H, Dohy Z, Czimbalmos C, Merkely B, Boldin-Adamsky S, Feinstein E, Horvath IG, Ferdinandy P (2021). Post-myocardial infarction heart failure in closed-chest coronary occlusion/reperfusion model in Gottingen Minipigs and landrace pigs. J Vis Exp.

[5] Cour M, Lecour S (2019). Remote ischaemic conditioning: in search of a suitable match. Nat Rev Cardiol.

[6] Danos O, Davies K, Lehn P, Mulligan R (2010). The ARRIVE guidelines, a welcome improvement to standards for reporting animal research. J Gene Med.

[7] de Waha S, Patel MR, Granger CB, Ohman EM, Maehara A, Eitel I, Ben-Yehuda O, Jenkins P, Thiele H, Stone GW (2017). Relationship between microvascular obstruction and adverse events following primary percutaneous coronary intervention for ST-segment elevation myocardial infarction: an individual patient data pooled analysis from seven randomized trials. Eur Heart J.

[8] Ferdinandy P, Baczko I, Bencsik P, Giricz Z, Gorbe A, Pacher P, Varga ZV, Varro A, Schulz R (2019). Definition of hidden drug cardiotoxicity: paradigm change in cardiac safety testing and its clinical implications. Eur Heart J.

[9] Ferdinandy P, Hausenloy DJ, Heusch G, Baxter GF, Schulz R (2014). Interaction of risk factors, comorbidities, and comedications with ischemia/reperfusion injury and cardioprotection by preconditioning, postconditioning, and remote conditioning. Pharmacol Rev.

[10] Hausenloy DJ, Botker HE, Engstrom T, Erlinge D, Heusch G, Ibanez B, Kloner RA, Ovize M, Yellon DM, Garcia-Dorado D (2017). Targeting reperfusion injury in patients with ST-segment elevation myocardial infarction: trials and tribulations. Eur Heart J.

[11] Hausenloy DJ, Chilian W, Crea F, Davidson SM, Ferdinandy P, Garcia-Dorado D, van Royen N, Schulz R, Heusch G (2019). The coronary circulation in acute myocardial ischaemia/reperfusion injury: a target for cardioprotection. Cardiovasc Res.

[12] Hausenloy DJ, Heusch G (2019). Translating cardioprotection for patient benefit: the EU-CARDIOPROTECTION COST Action. J Am Coll Cardiol.

[13] He Z, Davidson SM, Yellon DM (2020). The importance of clinically relevant background therapy in cardioprotective studies. Basic Res Cardiol.

[14] Heusch G (2018). Cardioprotection research must leave its comfort zone. Eur Heart J.

[15] Heusch G (2016). The coronary circulation as a target of cardioprotection. Circ Res.

[16] Heusch G (2019). Coronary microvascular obstruction: the new frontier in cardioprotection. Basic Res Cardiol.

[17] Heusch G (2017). Critical issues for the translation of cardioprotection. Circ Res.

[18] Heusch G (2020). Myocardial ischaemia-reperfusion injury and cardioprotection in perspective. Nat Rev Cardiol.

[19] Heusch G, Gersh BJ (2017). The pathophysiology of acute myocardial infarction and strategies of protection beyond reperfusion: a continual challenge. Eur Heart J.

[20] Heusch G, Libby P, Gersh B, Yellon D, Bohm M, Lopaschuk G, Opie L (2014). Cardiovascular remodelling in coronary artery disease and heart failure. Lancet.

[21] Heusch G, Skyschally A, Kleinbongard P (2018). Translation, translation, translation. Circ Res.

[22] Heusch G, Skyschally A, Schulz R (2011). The in-situ pig heart with regional ischemia/reperfusion—ready for translation. J Mol Cell Cardiol.

[23] Ho AFW, Chong J, Ong MEH, Hausenloy DJ (2020). Remote ischemic conditioning in emergency medicine-clinical frontiers and research opportunities. Shock.

[24] Hooijmans CR, de Vries R, Leenaars M, Curfs J, Ritskes-Hoitinga M (2011). Improving planning, design, reporting and scientific quality of animal experiments by using the Gold Standard Publication Checklist, in addition to the ARRIVE guidelines. Br J Pharmacol.

[25] Jones SP, Tang XL, Guo Y, Steenbergen C, Lefer DJ, Kukreja RC, Kong M, Li Q, Bhushan S, Zhu X, Du J, Nong Y, Stowers HL, Kondo K, Hunt GN, Goodchild TT, Orr A, Chang CC, Ockaili R, Salloum FN, Bolli R (2015). The NHLBI-sponsored Consortium for preclinicAl assESsment of cARdioprotective therapies (CAESAR): a new paradigm for rigorous, accurate, and reproducible evaluation of putative infarct-sparing interventions in mice, rabbits, and pigs. Circ Res.

[26] Kleinbongard P, Botker HE, Ovize M, Hausenloy DJ, Heusch G (2020). Co-morbidities and co-medications as confounders of cardioprotection-Does it matter in the clinical setting?. Br J Pharmacol.

[27] Kukreja R, Tang XL, Lefer D, Steenbergen C, Jones S, Guo Y, Li Q, Kong M, Stowers H, Hunt G, Tokita Y, Wu W, Ockaili R, Salloum F, Book M, Du J, Bhushan S, Goodchild T, Chang C, Bolli R (2014). Administration of sildenafil at reperfusion fails to reduce infarct size: results from the CAESAR Cardioprotection Consortium (LB650). FASEB J.

[28] Lecour S, Botker HE, Condorelli G, Davidson SM, Garcia-Dorado D, Engel FB, Ferdinandy P, Heusch G, Madonna R, Ovize M, Ruiz-Meana M, Schulz R, Sluijter JP, Van Laake LW, Yellon DM, Hausenloy DJ (2014). ESC working group cellular biology of the heart: position paper: improving the preclinical assessment of novel cardioprotective therapies. Cardiovasc Res.

[29] Lefer D, Jones S, Steenbergen C, Kukreja R, Guo Y, Tang XL, Li Q, Ockaili R, Salloum F, Kong M, Polhemus D, Bhushan S, Goodchild T, Chang C, Book M, Du J, Bolli R (2014). Sodium nitrite fails to limit myocardial infarct size: results from the CAESAR Cardioprotection Consortium (LB645). FASEB J.

[30] Lefer DJ, Bolli R (2011). Development of an NIH consortium for preclinicAl AssESsment of CARdioprotective therapies (CAESAR): a paradigm shift in studies of infarct size limitation. J Cardiovasc Pharmacol Ther.

[31] Perrino C, Ferdinandy P, Botker HE, Brundel B, Collins P, Davidson SM, den Ruijter HM, Engel FB, Gerdts E, Girao H, Gyongyosi M, Hausenloy DJ, Lecour S, Madonna R, Marber M, Murphy E, Pesce M, Regitz-Zagrosek V, Sluijter JPG, Steffens S, Gollmann-Tepekoylu C, Van Laake LW, Van Linthout S, Schulz R, Ytrehus K (2021). Improving translational research in sex-specific effects of comorbidities and risk factors in ischaemic heart disease and cardioprotection: position paper and recommendations of the ESC Working Group on Cellular Biology of the Heart. Cardiovasc Res.

[32] Ramirez FD, Motazedian P, Jung RG, Di Santo P, MacDonald ZD, Moreland R, Simard T, Clancy AA, Russo JJ, Welch VA, Wells GA, Hibbert B (2017). Methodological rigor in preclinical cardiovascular studies: targets to enhance reproducibility and promote research translation. Circ Res.

[33] Rossello X, Rodriguez-Sinovas A, Vilahur G, Crisostomo V, Jorge I, Zaragoza C, Zamorano JL, Bermejo J, Ordonez A, Bosca L, Vazquez J, Badimon L, Sanchez-Margallo FM, Fernandez-Aviles F, Garcia-Dorado D, Ibanez B (2019). CIBER-CLAP (CIBERCV Cardioprotection Large Animal Platform): a multicenter preclinical network for testing reproducibility in cardiovascular interventions. Sci Rep.

[34] Schwartz Longacre L, Kloner RA, Arai AE, Baines CP, Bolli R, Braunwald E, Downey J, Gibbons RJ, Gottlieb RA, Heusch G, Jennings RB, Lefer DJ, Mentzer RM, Murphy E, Ovize M, Ping P, Przyklenk K, Sack MN, Vander Heide RS, Vinten-Johansen J, Yellon DM, National Heart L, Institute B, NIoH, (2011). New horizons in cardioprotection: recommendations from the 2010 National Heart, Lung, and Blood Institute Workshop. Circulation.

[35] Stone GW, Selker HP, Thiele H, Patel MR, Udelson JE, Ohman EM, Maehara A, Eitel I, Granger CB, Jenkins PL, Nichols M, Ben-Yehuda O (2016). Relationship between infarct size and outcomes following primary PCI: patient-level analysis from 10 randomized trials. J Am Coll Cardiol.

[36] Szummer K, Wallentin L, Lindhagen L, Alfredsson J, Erlinge D, Held C, James S, Kellerth T, Lindahl B, Ravn-Fischer A, Rydberg E, Yndigegn T, Jernberg T (2017). Improved outcomes in patients with ST-elevation myocardial infarction during the last 20 years are related to implementation of evidence-based treatments: experiences from the SWEDEHEART registry 1995–2014. Eur Heart J.

